# An Actor-Partner Interdependence Mediation Model for Assessing the Association Between Health Literacy and mHealth Use Intention in Dyads of Patients With Chronic Heart Failure and Their Caregivers: Cross-Sectional Study

**DOI:** 10.2196/63805

**Published:** 2025-03-06

**Authors:** Xiaorong Jin, Yimei Zhang, Min Zhou, Qian Mei, Yangjuan Bai, Qiulan Hu, Wei Wei, Xiong Zhang, Fang Ma

**Affiliations:** 1 Department of General Surgery I, The People’s Hospital of ChuXiong Yi Autonomous Prefecture, ChuXiong, Yunnan Province, China; 2 Department of Nursing, The First Affiliated Hospital of Kunming Medical University, 295 Xichang Road, Kunming City, Yunnan Province, 650032, China, 86 65324888; 3 Coronary Heart Disease Center, Fuwai Yunnan Cardiovascular Hospital, Kunming City, Yunnan Province, China; 4 Cardiology Department, The First Affiliated Hospital of Kunming Medical University, Kunming City, Yunnan Province, China; 5 Department of Geriatric Intensive Medicine, The First Affiliated Hospital of Kunming Medical University, Kunming City, Yunnan Province, China; 6 Digestive Surgery Department, The First Affiliated Hospital of Kunming Medical University, Kunming City, Yunnan Province, China

**Keywords:** chronic heart failure, caregivers, health literacy, mHealth, actor-partner interdependence mediation model, mobile health

## Abstract

**Background:**

Chronic heart failure (CHF) has become a serious threat to the health of the global population. Self-management is the key to treating CHF, and the emergence of mobile health (mHealth) has provided new ideas for the self-management of CHF. Despite the many potential benefits of mHealth, public utilization of mHealth apps is low, and poor health literacy (HL) is a key barrier to mHealth use. However, the mechanism of the influence is unclear.

**Objective:**

The aim of this study is to explore the dyadic associations between HL and mHealth usage intentions in dyads of patients with CHF and their caregivers, and the mediating role of mHealth perceived usefulness and perceived ease of use in these associations.

**Methods:**

This study had a cross-sectional research design, with a sample of 312 dyads of patients with CHF who had been hospitalized in the cardiology departments of 2 tertiary care hospitals in China from March to October 2023 and their caregivers. A general information questionnaire, the Chinese version of the Heart Failure-Specific Health Literacy Scale, and the mHealth Intention to Use Scale were used to conduct the survey; the data were analyzed using the actor-partner interdependence mediation model.

**Results:**

The results of the actor-partner interdependent mediation analysis of HL, perceived usefulness of mHealth, and mHealth use intention among patients with CHF and their caregivers showed that all of the model’s actor effects were valid (*β*=.26‐0.45; *P*<.001), the partner effects were partially valid (*β*=.08‐0.20; *P*<.05), and the mediation effects were valid (*β*=.002‐0.242, 95% CI 0.003‐0.321; *P*<.05). Actor-partner interdependent mediation analyses of HL, perceived ease of use of mHealth, and mHealth use intention among patients with CHF and caregivers showed that the model’s actor effect partially held (*β*=.17‐0.71; *P*<.01), the partner effect partially held (*β*=.15; *P*<.01), and the mediation effect partially held (*β*=.355‐0.584, 95% CI 0.234‐0.764; *P*<.001).

**Conclusions:**

Our study proposes that the HL of patients with CHF and their caregivers positively contributes to their own intention to use mHealth, suggesting that the use of mHealth by patients with CHF can be promoted by improving the HL of patients and caregivers. Our findings also suggest that the perceived usefulness of patients with CHF and caregivers affects patients’ mHealth use intention, and therefore patients with CHF and their caregivers should be involved throughout the mHealth development process to improve the usability of mHealth for both patients and caregivers. This study emphasizes the key role of patients’ perception that mHealth is easy to use in facilitating their use of mHealth. Therefore, it is recommended that the development of mHealth should focus on simplifying operational procedures and providing relevant operational training according to the needs of the patients when necessary.

## Introduction

Heart failure is a condition of signs and symptoms triggered by structural or functional abnormalities of the heart, as evidenced by elevated natriuretic peptide levels and cardiogenic, pulmonary, or systemic congestion [1]. Chronic heart failure (CHF) manifests as a persistent state of heart failure that may be stable, worsening, or decompensated [2]. As a severe or advanced manifestation of a wide range of cardiovascular diseases, CHF is known for its high morbidity and mortality and has become an important public health problem threatening the health and lives of the global population [3]. According to the Global Heart Failure Survey, the average incidence of CHF reaches 460/100,000 persons/year, while the 5- and 10-year survival rates of patients after diagnosis are only 57% and 35%, respectively [4,5]. More worryingly, approximately 60% of patients with CHF die within 5 years of diagnosis [6,7].

Studies have shown that self-management is important for patients with CHF to improve cardiac function, improve disease prognosis, and reduce mortality [8]. With the increasing popularity of smartphones and wearable devices, mobile health (mHealth) has gradually become a new means to support self-management in patients with CHF [9]. mHealth refers to the integrated application of mobile phones and other wireless technologies in medical practice, and its functions cover a wide range of aspects such as booking appointments, health information inquiry, and vital signs monitoring [9]. Although the role of mHealth in facilitating self-management in patients with CHF has been widely recognized, the actual usage of mHealth by patients is generally low [10,11].

Health literacy (HL) refers to an individual’s ability to access, understand, process, and utilize health information to promote one’s health [12]. As information largely moves to a digital medium, HL takes on an added dimension in supporting the skills needed to understand online health information, which makes digital health a significant component of HL [13-15]. It has been evidenced that digital HL is an important indicator of mHealth use intention, indicating that HL is an important factor influencing mHealth use intention [16]. On the other hand, mHealth use intention would reinforce individual motivation and confidence to broadly develop their literacy about digital health and HL as well [17]. Additionally, the study indicates that individuals who intend to seek health-related information or advice on the internet exhibit higher digital HL levels, suggesting that mHealth use intention could contribute to an increase in HL [18]. Existing studies have shown that HL is positively associated with mHealth use intention and behavior. Specifically, the higher the level of HL of patients, the more likely they are to adopt and use mHealth technologies, and vice versa [19,20]. Although the association between HL and willingness to use mHealth has been reported several times, the exact mechanism remains to be further explored. According to the Technology Acceptance Model (TAM) theory, users’ actual adoption or rejection of a technology such as mHealth is primarily affected by 2 factors, namely perceived usefulness and perceived ease of use [21]. The same researchers determined that perceived ease of use and perceived usefulness of telemedicine services have a substantial effect on the telemedicine behavioral intention of older patients [21]. Additionally, it has been evidenced that HL can influence the perceived usefulness and perceived ease of use of mHealth [22,23]. Hence, perceived usefulness and perceived ease of use of mHealth might act as mediators of HL and mHealth use intention.

In addition, previous studies have pointed out that caregivers play an integral role in the self-management of patients with CHF [24,25]. Cheng et al suggested that caregivers bear 70% of the responsibility for chronic disease management [26]. According to Interdependence Theory, in a dyadic relationship, each person has the ability to influence the outcomes of the other, suggesting partners affect each other’s motives, preferences, behaviors, and health outcomes [27,28]. Patients with CHF and their caregivers constitute a dyad, and the attributes of one dyad member can influence the motive and behavior of the other [26], hence, patients’ and caregivers’ HL might influence the partners’ mHealth use intentions. The social influence variable in the Unified Theory of Acceptance and Use of Technology model also suggests that a patient’s willingness to adopt a technology may be influenced by the perceptions of important people around them, such as caregivers [29,30]. However, current research has mostly explored mHealth use intentions from the single perspective of either the patient or the caregiver, ignoring the interactions between the two.

Therefore, this study explored in depth the underlying mechanism linking HL and mHealth use intention of patients with CHF and their caregivers from a dyad perspective, which can help provide targeted intervention for future mHealth use intention studies, thus promoting the use of mHealth among patients with CHF.

## Methods

### Design and Sample

This study adopted a cross-sectional research design with a convenience sampling method. Patients with CHF admitted to the cardiology departments of 2 tertiary care hospitals in Yunnan Province, China, from March to October 2023 and their caregivers were selected for the study. The inclusion criteria for patients with CHF were as follows: (1) age ≥18 years old; (2) compliance with the 2022 AHA/ACC/HFSA Guidelines for the Management of Heart Failure [2] diagnostic criteria for CHF; (3) meeting New York Heart Association (NYHA) cardiac function class I-III; (4) at least one caregiver; (5) being able to complete the questionnaire independently or under the guidance of the researchers; and (6) voluntarily participating in this study. The exclusion criteria for patients with CHF were as follows: (1) acute myocardial infarction or acute pulmonary embolism in the past month; (2) with other critical diseases, such as malignant tumor, renal failure, respiratory failure; (3) not using smartphones; (4) cognitive disorders or psychiatric disorders; and (5) being involved in other research projects. Caregiver inclusion criteria were as follows: (1) informal caregivers who are the primary caregiver and provide care and support to the patient without compensation [26,31]; (2) age ≥18 years old; (3) being able to complete the questionnaire independently or under the guidance of the researchers; and (4) voluntarily participating in the study. Caregiver exclusion criteria were as follows: (1) cognitive impairment or mental illness; (2) serious physical disease, such as cancer or vital organ failure; (3) not using smartphones; and (4) participating in other studies. According to Ledermann et al [32], in the actor-partner interdependence model (APIM), a sample size of approximately 93 to 241 dichotomies is recommended, while for the actor-partner interdependence mediation model (APIMeM), a sample size of approximately 120 dichotomies is required for good mediation. Of 367 eligible patient-caregiver dyads, 31 were excluded from enrollment because one member of the dyad refused to participate, and 24 were excluded from the analysis because of missing data. Thus, 312 dyads were included in the final data analysis. This sample size met the requirement for structural equation modeling and APIMeM.

### Data Collection

Before the survey, all of the researchers received unified training to familiarize themselves with the content of the questionnaire scale and to master the way of filling it out and the precautions to be taken. Data collection was carried out by patients with CHF and their caregivers alone, with the researcher providing guidance but not interfering with the study subjects. After the survey was completed, researchers checked the data on the spot; if they found any missing or wrongly filled items, they verified the data with the research subjects immediately and assisted them in completing the data.

### Measures

#### Demographic and Clinical Characteristics

A questionnaire, which was designed by the researchers based on a large amount of literature, was used in the survey, and it included patients’ information such as gender, age, literacy level, marital status, smoking history, drinking history, duration of heart failure, number of hospitalizations, NYHA cardiac function classification, previous history of cardiovascular disease, left ventricular ejection fraction, and BMI, as well as the caregiver’s information such as gender, age, relationship with the patient, education level, occupation, marital status, monthly income, total caregiving time, caregiving time per day, and illness.

#### Health Literacy

The Heart Failure–Specific Health Literacy Scale (HF-SHLS) was used to measure HL; it was initially developed by Matsuoka et al [33] and Chineseized by Yue Meng et al [34] in 2016. The HF-SHLS consists of 3 dimensions with 12 entries, namely functional HL, interactive HL, and critical HL; each entry ranks from “not applicable” (score of 1) to “very applicable” (score of 4), and the total score ranges between 12‐48, with a higher score representing a higher level of HL. The Cronbach values of the overall scale and 3 dimensions were 0.87, 0.84, 0.72, and 0.69, respectively [33,34], with acceptable internal consistency, and the scale was used with permission from the original authors.

#### mHealth Use Intention

In this study, “mHealth” refers to the integrated application of smartphones in medical practice, including activities such as booking appointments and inquiring about health information. The mHealth Service Use Intention Questionnaire, which was prepared by Shi Lin [35] was used to measure the perceived usefulness of mHealth, perceived ease of use of mHealth, and mHealth use intentions, which consists of 7 dimensions and 26 entries. Cronbach values for each dimension ranged from 0.822‐0.968. In this study, the 3 dimensions measuring the perceived usefulness of mHealth, perceived ease of use of mHealth, and mHealth use intentions were used, with Cronbach values of each dimension being 0.904, 0.846, and 0.968, respectively. The indicators in the questionnaire are scored on a 5-point Likert scale, with scores of 1‐5 representing strongly disagree, disagree, undecided, agree, and strongly agree; a higher score in the related dimension means a higher level of perceived usefulness of mHealth, perceived ease of use of mHealth, and mHealth use intentions. The scale was used with permission from the original authors. For the detailed items and response options of this questionnaire, please refer to Multimedia Appendix 1.

### Statistical Analyses

SPSS (version 26.0; IBM Corp) and AMOS (version 26.0; IBM Corp) were used for data analysis. A value of *P*<.05 based on a 2-tailed test was considered statistically significant. Data were tested for normality (results are detailed in Multimedia Appendix 2), which was based on the Kolmogorov-Smirnov test when the sample size was greater than 50, and when *P*<.05, it was considered as not conforming to normal distribution [36]. The results showed that the data in this study did not conform to normal distribution. Thus, we used median and interquartile spacing to describe the skewed distribution. The Wilcoxon signed-rank sum test was used to detect differences in scores for HL, perceived usefulness of mHealth, perceived ease of use of mHealth, and mHealth use intention among patients with CHF and their caregivers. Spearman correlation analysis was used to test the relationship between HL, mHealth perceived usefulness, and perceived ease of mHealth in the patient-caregiver dyad in the context of CHF. APIMeM was used to construct the APIMeM of HL-perceived usefulness of mHealth-intention to use mHealth in patients with CHF and their caregivers and the APIMeM of HL-perceived ease of use of mHealth-mHealth use intention in patients with CHF and their caregivers. The nonnormal distributions were calculated using the bootstrap method. The fit of each model was assessed using *Χ*^2^/*df* (<3), root mean square error of approximation (RMSEA; ≤0.08), comparative fit index (CFI; ≥0.90), and Tucker-Lewis index (TLI; ≥0.90). The mediating effects in APIMeM were tested using bootstrap analyses, with a sample size of 5000 and a confidence interval of 95%. Furthermore, we conducted multigroup APIMeM to analyze whether age and relationship with the patient moderated the dyadic effects. We used a *t* test, Mann-Whitney test, or Kruskal-Wallis test to determine whether there was a significant difference in the path coefficients between groups based on the normality of the path coefficients of each group; significance tests were set at a 2-sided, *P*≤.05.

### Ethical Considerations

The study was approved by the Ethics Committees of the First Affiliated Hospital of Kunming Medical University (2022-L-304) and Fuwai Cardiovascular Disease Hospital of Yunnan Province (2023-033-01) and was conducted in accordance with the principles of the Declaration of Helsinki. Informed consent was obtained from all participants before the investigation. Participation in the study was entirely voluntary, and no compensation was provided for their involvement. Participant data were deidentified and stored on a local secure server. Further, the participants had the right to withdraw from the study at any time, without giving any reasons. In addition, no identification of individual participants or users in any images occurred in the manuscript or supplementary material.

## Results

### Characteristics of the Patient-Caregiver Dyads

A total of 312 dyads of patients with CHF and their caregivers were included in the final analysis. The age of patients with CHF ranged from 18 to 89 years old; most of them were male (65.7%), their education level was primary school or below (32%), their NYHA classification was mostly grade II (46.8%), 130 of them had a left ventricular ejection fraction of more than 50% (41.7%), and almost half of the patients’ BMIs were in the range of 18.5‐23.9 (49%). The age of caregivers ranged from 18 to 84 years old; most of them were female (62.2%), their education level was mainly junior high school (25.6%) and bachelor’s degree (27.6%), and most of them were the children of patients (46.6%); for 41.35% of the caregivers, the average per capita monthly income was 2000‐4999 yuan (US $274-$686), and for most caregivers, the total time of caregiving was less than 1 year (76%). Detailed information is shown in Table 1.

**Table 1.** General demographic and disease-related information for patients with chronic heart failure and their caregivers.

Variable and clusters		Patients with chronic heart failure (N=312）		Caregivers (N=312）	
		Frequency	Percentage	Frequency	Percentage
**Sex**					
	Male	205	65.7	118	37.8
	Female	107	34.3	194	62.2
**Age group (years)**					
	18‐44	41	13.1	152	48.7
	45‐59	114	36.5	111	35.6
	60‐74	107	34.3	39	12.5
	75‐89	50	16	10	3.2
**Education**					
	≤Primary school	100	32	32	10.3
	Junior high school	86	27.6	80	25.6
	Senior high school	48	15.4	51	16.3
	Associate degree	33	10.6	55	17.6
	Bachelor’s degree	41	13.1	86	27.6
	≥Master’s degree	4	1.3	8	2.6
**New York Heart Association classification**					
	I	43	13.8	—^a^	—
	II	146	46.8	—	—
	III	123	39.4	—	—
**Left ventricular ejection fraction**					
	<30	55	17.6	—	—
	30‐50	127	40.7	—	—
	>50	130	41.7	—	—
**BMI**					
	<18.5	25	8	—	—
	18.5‐23.9	153	49	—	—
	24.0‐27.9	93	29.8	—	—
	≥28.0	41	13.1	—	—
**Relationship of caregiver to patient**					
	Parents	—	—	8	2.6
	Spouse	—	—	131	42.0
	Son/daughter	—	—	144	46.2
	Friend	—	—	6	1.9
	Other kinships	—	—	23	7.4
**Monthly per capita income (yuan)^b^**					
	2000	—	—	64	20.5
	2000‐4999	—	—	129	41.3
	≥5000	—	—	119	38.1
**Duration of caregiving (years)**					
	<1	—	—	237	76.0
	1‐2.9	—	—	23	7.4
	3‐5.9	—	—	10	3.2
	≥6	—	—	42	13.5

a Not applicable.

b A currency exchange rate of 1 yuan=US $0.14 is applicable.

### The Scores and Differences in the Study Variables in the Dyadic Group

The median HL score of patients with CHF was 34.00 (IQR27.00-40.00), which was lower than that of their caregivers (median 37.00, IQR 31.00-42.00), with a statistically significant difference between them (*P*<.001). The median (IQR) scores of perceived usefulness, perceived ease of use, and mHealth use intention in patients with CHF were 20.00 (17.00-23.00), 12.00 (6.00-15.00), and 14.00 (11.00-15.00), respectively; the median (IQR) perceived usefulness, perceived ease of use, and mHealth use intention in caregivers were 21.00 (19.00-25.00), 15.00 (12.00-15.00), and 15.00 (14.00-15.00), respectively. The patients’ scores were lower than those of carers, with statistically significant differences between them (*P*<.001), as shown in Table 2.

**Table 2.** Scores and differences in variables among patients with chronic heart failure and their caregivers.

Dimension	Patients with chronic heart failure (N=312), median (IQR)	Caregivers (N=312), median (IQR)	*Z*-score	*P* value
Health literacy	34.00 (27.00-40.00)	37.00 (31.00-42.00）	4.90	<.001
Perceived usefulness	20.00 (17.00-23.00)	21.00 (19.00-25.00）	4.38	<.001
Perceived ease of use	12.00 (6.00-15.00)	15.00 (12.00-15.00）	7.88	<.001
Mobile health use intentions	14.00 (11.00-15.00)	15.00 (14.00-15.00）	6.86	<.001

### Correlation Coefficients of Study Variables

Among patients with CHF, mHealth use intention was positively correlated with their own HL, mHealth perceived usefulness, and perceived ease of use (*r*=0.512‐0.682, *P*<.05), and positively correlated with the caregiver’s mHealth perceived usefulness and mHealth use intention (*r*=0.222‐0.310, *P*<.05); caregiver’s mHealth use intention was positively correlated with their own HL, mHealth perceived usefulness, and perceived ease of use (*r*=0.393‐0.572, *P*<.01), and positively correlated with patients’ HL, mHealth perceived usefulness, perceived ease of use, and mHealth use intention (*r*=0.170‐0.310, *P*<.01). The results of the correlation analyses are detailed in Table 3.

**Table 3.** Correlations between health literacy, perceived usefulness, perceived ease of use, and mobile health use intentions in patients with chronic heart failure and their caregivers.

Variable	1^a^	2^b^	3^c^	4^d^	5^e^	6^f^	7^g^	8^h^
1	1							
2	0.432^i^	1						
3	0.717^i^	0.465^i^	1					
4	0.577^i^	0.512^i^	0.682^i^	1				
5	0.211^i^	0.089	0.142^j^	0.094	1			
6	0.144^j^	0.361^i^	0.132^j^	0.222^i^	0.327^i^	1		
7	0.124^j^	0.164^i^	0.130^j^	0.109	0.466^i^	0.438^i^	1	
8	0.170^i^	0.282^i^	0.204^i^	0.310^i^	0.393^i^	0.527^i^	0.572^i^	1

a Health literacy of patients with chronic heart failure.

b Patients’ perceived usefulness of mobile health.

c Patients’ perceived ease of use of mobile health.

d Patients’ mobile health use intention.

e Caregivers’ health literacy.

f Caregivers’ perceived usefulness of mobile health.

g Caregivers’ perceived ease of use of mobile health.

h Caregivers’ mobile health use intention.

i *P<*.01.

j *P*<.05.

### The APIMeM of HL-Perceived Usefulness of mHealth-mHealth Use Intention in Patients With CHF and Caregivers

In this study, the model fit index values were *Χ*^2^ (*df*)=1.175 (<3), RMSEA=0.024 (≤0.08), CFI=0.992 (≥0.90), TLI=0.995 (≥0.90), which indicated that the model was well fitted.

### Direct Effect Analysis

**Figure 1.**
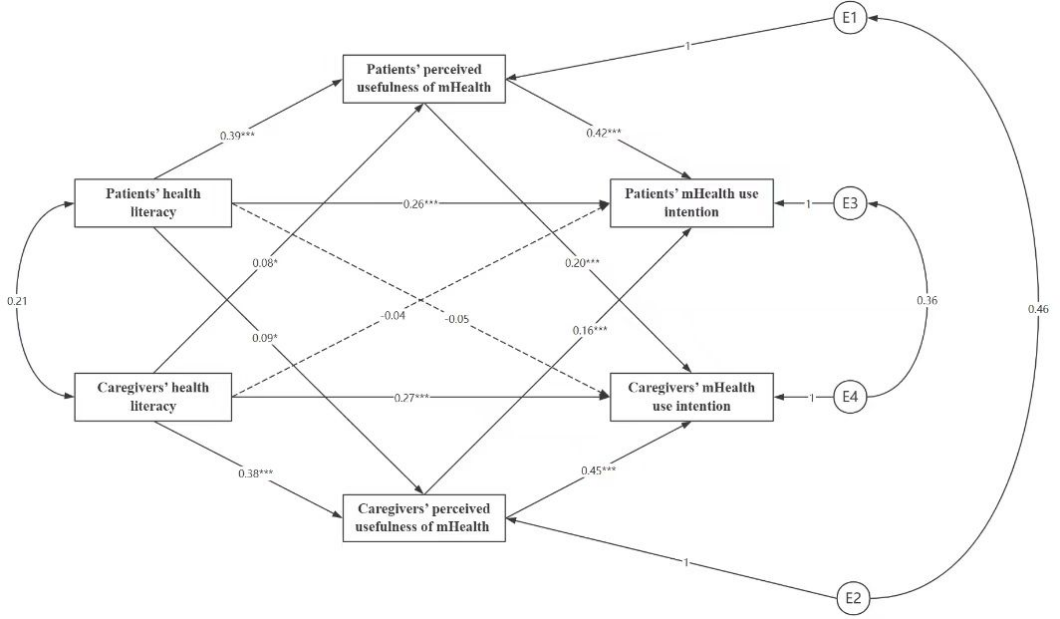
APIMeM results of health literacy and perceived usefulness of mHealth on mHealth use intention in patient-caregiver dyads. Dashed lines indicate insignificant path coefficients and solid lines indicate significant path coefficients (*P<.05, **P<.01, ***P<.001). APIMeM: actor-partner interdependence mediation model; mHealth: mobile health.

The results showed that (1) patients’ and caregivers’ HL was a positive predictor of their own perceived usefulness of mHealth (*β*=.38‐0.39; *P*<.001); (2) patients’ and caregivers’ perceived usefulness of mHealth was a positive predictor of their own mHealth use intention (*β*=.42‐0.45; *P*<.001); (3) patients’ and caregivers’ HL had a positive predictive effect on their own mHealth use intention (*β*=.26‐0.27; *P*<.001), that is, the subject effects of the model all held; (4) HL of patients and caregivers had a positive predictive effect on each other’s perceived usefulness of mHealth (*β*=.08‐0.09; *P*<.05); (5) perceived usefulness of mHealth of patients and caregivers had a positive predictive effect on each other’s mHealth use intention (*β*=.16‐0.20; *P*<.001); (6) patients’ and caregivers’ HL had a nonsignificant effect on each other’s mHealth use intention, that is, the object effect was partially established (see Figure 1 for details).

### Mediation Analysis

Bootstrap tests for mediating effects showed the patients’ and caregivers’ HL directly positively predicted their own mHealth use intention (*β*=.381, 95% CI 0.279‐0.495; *P*<.001) and also positively predicted both their own (*β*=.242, 95% CI 0.171‐0.321; *P*<.001) and their counterpart’s mHealth perceived usefulness (*β*=.022, 95% CI 0.003‐0.052*; P*=.02), which meant that the perceived usefulness of mHealth for patients with CHF and caregivers played a partial mediating role between their own and their counterpart’s HL and mHealth use intention. In addition, patients’ and caregivers’ HL did not directly predict their counterpart’s mHealth use intention (*β*=−.068, 95% CI −0.168‐0.038; *P*=.21), but it could be positively predicting own perceived usefulness (*β*=.102, 95% CI 0.049‐0.167; *P*<.001) and their counterpart’s perceived usefulness (*β*=.053, 95% CI 0.006‐0.107; *P*=.02), further positively predicting their own intention to use mHealth and their counterpart’s intention to use mHealth, which meant that mHealth perceived usefulness of patients with CHF and caregivers played a fully mediating role in the relationship between their own HL and their counterpart’s mHealth use intention (see Table 4 for details).

**Table 4.** The direct, indirect, and total indirect effects for patients with chronic heart failure and caregivers in the actor-partner interdependence mediation model (N=312 dyads).

	*β* ^a^	SE	95% CI	*P* value
**Actor effect**				
***Patients***				
Total indirect effect	.264	0.046	0.182 to 0.356	<.001
1^b^HL^c^→1PU^d^→1UI^e^	.242	0.039	0.171 to 0.321	<.001
1HL→2^f^ PU→1 UI	.022	0.012	0.003 to 0.052	.02
Direct effect	.381	0.054	0.279 to 0.495	<.001
***Caregivers***				
Total indirect effect	.264	0.046	0.182 to 0.356	<.001
2HL→1PU→2 UI	.022	0.012	0.003 to 0.052	.02
2HL→2PU→2 UI	.242	0.039	0.171 to 0.321	<.001
Direct effect	.381	0.054	0.279 to 0.495	<.001
**Partner effect**				
***Patients***				
Total indirect effect	.154	0.044	0.072 to 0.245	<.001
2HL→1PU→1 UI	.053	0.026	0.006 to 0.107	.02
2HL→2PU→1 UI	.102	0.030	0.049 to 0.167	<.001
Direct effect	−.068	0.052	−0.168 to 0.038	.21
***Caregivers***				
Total indirect effect	.154	0.044	0.072 to 0.245	<.001
1HL→1PU→2 UI	.102	0.030	0.049 to 0.167	<.001
1HL→2PU→2 UI	.053	0.026	0.006 to 0.107	.02
Direct effect	−.068	0.052	−0.168 to 0.038	.21

a *β*: standardized estimate.

b 1: patients.

c HL: health literacy.

d PU: perceived usefulness.

e UI: use intention.

f 2: caregivers.

### The APIMeM of HL-Perceived Ease of Use of mHealth-mHealth Use Intention in Patients With CHF and Caregivers

The model was a saturated model with 0 degrees of freedom [37], so its fit indices would no longer be estimated and only its path coefficients would be of interest [38].

**Figure 2.**
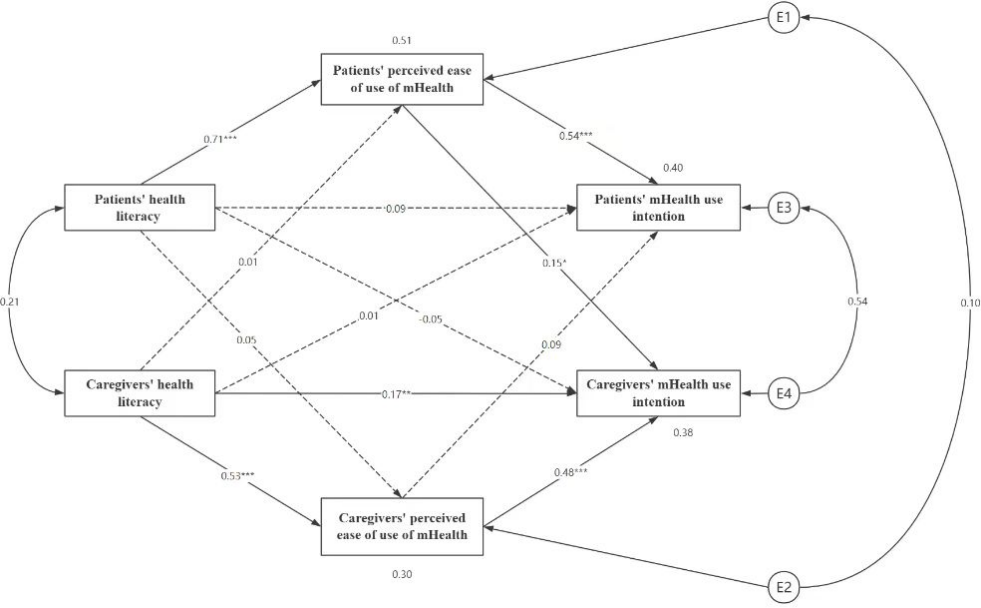
APIMeM results of health literacy and perceived ease of use on mHealth use intention in patient-caregiver dyads. Dashed lines indicate insignificant path coefficients and solid lines indicate significant path coefficients (*P<.05, **P<.01, ***P<.001). APIMeM: actor-partner interdependence mediation model; mHealth: mobile health.

The results showed that (1) patients’ and caregivers’ HL was a positive predictor of their own perceived ease of use (*β*=.53‐0.71; *P*<.001); (2) patients’ and caregivers’ perceived ease of use was a positive predictor of their own mHealth use intention (*β*=.48‐0.54; *P*<.001); (3) caregivers’ HL had a positive predictive effect on their own mHealth use intention (*β*=.17*; P*<.01), that is, the main effect of the model was partially established; and (4) perceived ease of use in patients had a positive predictive effect on the caregiver’s mHealth use intention (*β*=.15; *P*<.01); the rest of the object effects were not significant (see Figure 2 for details).

### Mediation Analysis

Bootstrap tests of mediating effects were conducted, and the results showed that patients’ HL positively predicted their own perceived ease of use, which then positively predicted their own mHealth use intention (*β*=.584, 95% CI 0.443‐0.764; *P*<.001), with perceived ease of use playing a fully mediating role. Caregivers’ HL directly positively predicted their own mHealth use intention (*β*=.235, 95% CI 0.079‐0.402*; P*=.003), as well as positively predicting their own perceived ease of use, which predicted their mHealth use intention (*β*=.355, 95% CI 0.234‐0.504; *P*<.001), and perceived ease of use played a partial mediating role. In addition, patients’ HL positively predicted their own perceived ease of use, which positively predicted their counterpart’s mHealth use intention (*β*=.128, 95% CI 0.012‐0.281; *P*=.02; see Table 5 for details).

**Table 5.** The direct, indirect, and total indirect effects for patients with chronic heart failure and caregivers in the actor-partner interdependence mediation model (N=312 dyads).

	*β* ^a^	SE	95% CI	*P* value
**Actor effect**				
***Patients***				
Total indirect effect	.591	0.082	0.449 to 0.775	<.001
1^b^HL^c^→1PEOU^d^→1UI^e^	.584	0.081	0.443 to 0.764	<.001
1HL→2^f^PEOU→1UI	.007	0.010	−0.004 to 0.043	.19
Direct effect	.138	0.087	−0.036 to 0.307	.11
***Caregivers***				
Total indirect effect	.357	0.071	0.232 to 0.509	.23
2HL→1PEOU→2UI	.002	0.008	−0.013 to 0.020	.63
2HL→2PEOU→2UI	.355	0.069	0.234 to 0.504	<.001
Direct effect	.235	0.082	0.079 to 0.402	.003
**Partner effect**				
***Patients***				
Total indirect effect	.089	0.073	−0.043 to 0.243	.19
2HL→1PEOU→1UI	.008	0.035	−0.060 to 0.080	.79
2HL→2PEOU→1UI	.082	0.064	−0.036 to 0.215	.17
Direct effect	.024	0.093	−0.160 to 0.205	.78
***Caregivers***				
Total indirect effect	.159	0.074	0.033 to 0.323	.01
1HL→1PEOU→2UI	.128	0.066	0.012 to 0.281	.02
1HL→2PEOU→2UI	.031	0.030	−0.023 to 0.094	.26
Direct effect	−.063	0.087	−0.244 to 0.095	.48

a *β*: standardized estimate.

b 1: patients.

c HL: health literacy.

d PEOU: perceived ease of use.

e UI: use intention.

f 2: caregivers.

### Moderated Effects of Age and Patient-Caregiver Relationship on Dyadic Effects

We regrouped the data into older and younger groups based on patient and caregiver age, and into spouse, child, and other groups based on patient-caregiver relationship, and used multigroup APIMeM to explore further whether the dichotomous effects of HL, perceived usefulness of mHealth, and perceived ease of use on mHealth use intention were moderated by patient and caregiver age and relationship. The results showed that the path coefficients of the APIMeM were not significantly different across groups (*P*>.05), meaning that no moderate influence of patient and caregiver age and relationship on the dichotomous effects of HL and mHealth perceived usefulness and perceived ease of use on mHealth use intention was observed. The details are shown in Multimedia Appendix 3.

## Discussion

### Principal Findings

The study took place during the heart of the COVID-19 pandemic, which forced much of the world to rely on eHealth and mHealth during this time. Some studies have reported an increase in internet dependence as a result of the COVID-19 pandemic, which has led to an increase in digital connectivity among older adults, thereby increasing the feasibility of their use of mHealth services [39]. Our study showed that the HL, perceived usefulness, perceived ease of use, and mHealth use intention of patients with CHF were significantly lower than caregivers’ scores, which might be due to the following reasons. First, most of the caregivers in the study were the children of the patients and were therefore younger. It has been evidenced that bodily functions and cognitive capacities deteriorate with age, resulting in decreased vision flexibility and memory loss, among other effects [40,41]. In addition, the younger generation, called “digital natives,” had earlier and more frequent access to the internet, whereas the ability of older adults to adapt to modern digital life wanes over time, and this “digital divide” of older adults means that they face greater difficulties and obstacles in the process of using mHealth [42]. Furthermore, previous studies have pointed out that HL declines with age [43]. Therefore, compared with younger caregivers, patients with CHF have more difficulties grasping and using mHealth, and they may show lower perceived usefulness, perceived ease of use, mHealth use intention, and HL as well. Second, research has shown that [44] physical limitations associated with illness might impede an individual’s ability to use technology, making it more arduous and challenging to use mHealth. The more severe the condition, the more likely the individual is to perceive more impediments to the use of mHealth [45]; therefore, compared with caregivers, people with CHF might exhibit lower levels of perceived usefulness, perceived ease of use, and mHealth use intention.

Our study showed that the HL of patients with CHF and their caregivers positively contributed to their own mHealth use intention. HL has emerged as a powerful predictor of self-care behaviors including mHealth use in the context of chronic illness, which can empower individuals to navigate the adoption of mHealth use [46]. HL entails people’s motivation to understand and apply health information in order to make decisions in everyday life to maintain or improve quality of life [47]; therefore, people with higher HL might be more motivated to use mHealth to manage disease. Evidence also suggests that individuals with low HL face greater challenges in computer use and reading, manipulating, and assessing health information [48], whereas individuals with higher levels of HL are more likely to comprehend and use mHealth [49], leading to a high level of mHealth use intention. Our study also found that the HL of patients with CHF and their caregivers positively influenced their counterparts’ mHealth use intention by the fully mediating role of perceived usefulness. Therefore, to improve patients’ mHealth use, not only is the improvement of their HL necessary but their caregivers’ enhanced HL is also highlighted.

Perceived usefulness was identified as a partial mediator of HL and mHealth use intention in patients with chronic heart disease and their caregivers in our study. This aligns with the broader literature emphasizing the importance of perceived usefulness in shaping mHealth adoption intention. As an important attribute of HL, reading skills entail a multitude of complex cognitive processes that require the ability to obtain meaning from the text being read. Additionally, HL lies in being able to use the information to make appropriate health care decisions [50]. All of the above suggests that higher HL might facilitate the understanding of mHealth, including the design, purpose, precautions, and adoption, leading to a higher level of perceived usefulness. It has also been evidenced by Wu et al [22,23] that HL positively affects the perceived usefulness of mHealth. Furthermore, usability issues, such as lack of an easy overview and nonintuitive app design, text, buttons, and icon elements, have been reported to be negatively correlated with mHealth utilization [51,52]. In addition, TAM theory also supports the positive relationship between perceived usefulness and mHealth use intention [53]. It was also suggested by the study of Dunn Lopez et al that mHealth apps must be readable, provide useful functions, and be based on evidence to improve patients’ intention to use mHealth [54]. We also identified that the perceived usefulness of patients acted as the full mediator of patients’ HL and caregivers’ mHealth use intention, and the perceived usefulness of caregivers acted as the full mediator of caregivers’ HL and patients’ mHealth use intention. The findings suggest that the perceived usefulness of the patients and caregivers can both influence the mHealth use intention of patients, highlighting that patients and caregivers should be involved during the redevelopment process of mHealth to improve its usability from the perspectives of patients and caregivers. The app content must be readable, provide useful functions, and be based on evidence.

In our study, perceived ease of use was identified as a partial mediator of HL and mHealth use intention in patients with chronic heart disease, and it was a full mediator of HL and mHealth use intention in caregivers. HL includes individuals’ capabilities to access and use available resources within the health care system, which allows individuals to increase their abilities to use preventive health services including mHealth to manage their health [55]. Thus, people with higher HL will show increased perceived ease of use of mHealth. In addition, perceived ease of use influencing individuals’ mHealth use intention is also attested by other researchers and is supported by TAM theory. Patients’ and caregivers’ concerns about mHealth apps being difficult and time-consuming to use, along with being not easy to access through the use of complex keywords, led to their unwillingness to use mHealth [56]. The actor-partner analysis showed that perceived ease of use of the patients was a full mediator of patients’ HL and their caregivers’ mHealth use intention; however, perceived ease of use of the caregivers was not a mediator of caregivers’ or patients’ HL and patients’ mHealth use intention. Therefore, caregivers’ perceived ease of use of mHealth will not induce patients’ willingness to adopt mHealth, and patients will choose to use it based on their own experience, highlighting the key role of perceived ease of use of the patients in facilitating their mHealth use. Nevertheless, some patients with CHF have expressed difficulty in downloading electronic health–related equipment and procedures and in operating health-monitoring equipment due to their complexity, which has increased their anxiety about its use [57]. Other research has also pointed out that the target population of mHealth expressed the desire for a simple and intuitive-to-use mobile app with nonmedical language and easy-to-understand material [58]. Therefore, it is recommended that the development of mHealth should focus on simplifying operational procedures and providing relevant operational training when necessary based on the needs of patients.

### Limitations

This study also has limitations. First, it was not possible to infer a causal relationship between the variables as this was a cross-sectional study. Second, this study only focused on the mediating effects of 2 variables, perceived usefulness and perceived ease of use of mHealth, and the mediation of the relationship between HL and mHealth use intention, ignoring other possible variables. To better explain how HL affects mHealth use intentions in the patient-caregiver dyad, future research should focus on other possible mediating or moderating variables. In addition, the increase in internet and digital technology use spurred on by the pandemic might have impacted the findings; therefore, our study findings should be considered with caution. Third, participants were from only 2 tertiary hospitals in Kunming, Yunnan Province, China, and most of the patient-caregiver relationships were between parents and children, who had a certain age gap. Therefore, the population characteristics and cultural differences between countries and regions may limit the generalizability of our findings.

### Conclusions

Our study proposes that patients with CHF will exhibit lower HL, perceived usefulness, perceived ease of use, and intention to use mHealth compared to their caregivers. Furthermore, the HL of patients with CHF and their caregivers positively contributes to their own mHealth use intention, with the full mediating effect of perceived usefulness. Therefore, mHealth utilization among patients with CHF could be promoted by improving their own HL and that of their caregivers as well. The findings suggest that both patients’ and caregivers’ perceived usefulness affects patients’ mHealth use intention, and therefore patients and caregivers should be involved in the entire mHealth development process in order to improve the usability of mHealth for patients and caregivers. The study highlights the key role of patients’ perception of mHealth as easy to use in facilitating their use of mHealth. Therefore, it is recommended that the development of mHealth should focus on simplifying operational procedures and providing relevant training about operations according to the needs of the patients when necessary.

## Supplementary material

10.2196/63805 Multimedia Appendix 1 Mobile Health Service Use Intention Questionnaire.

10.2196/63805 Multimedia Appendix 2 The results of the normality test.

10.2196/63805 Multimedia Appendix 3 The results of the multigroup actor-partner interdependence mediation model.

## Abbreviations

APIM: actor-partner interdependence model
APIMeM: actor-partner interdependence mediation model
CFI: comparative fit index
CHF: chronic heart failure
HF-SHLS: Heart Failure–Specific Health Literacy Scale
HL: health literacy
mHealth: mobile health
RMSEA: root mean square error of approximation
TAM: Technology Acceptance Model
TLI: Tucker-Lewis index
