# Supplementary material for: An Actor-Partner Interdependence Mediation Model for Assessing the Association Between Health Literacy and mHealth Use Intention in Dyads of Patients With Chronic Heart Failure and Their Caregivers: Cross-Sectional Study
Source: JMIR Mhealth Uhealth. 2025 Mar 6;13:e63805. doi: 10.2196/63805 (PMC11905925; doi:10.2196/63805)
Supplement: Multimedia Appendix 1 [file mhealth-v13-e63805-s001.pdf]

mHealth Service Use Intention Questionnaire

Dimension	Items	Strongly Disagree	Disagree	Uncertain	Agree	Strongly Agree
Perceived Usefulness	1. Using mobile health services saves me time.	1	2	3	4	5
	2. Using mobile health services improves my medical treatment efficiency.	1	2	3	4	5
	3. For sensitive health issues, it's easier to consult a doctor using mobile health services.	1	2	3	4	5
	4. Using mobile health services helps me obtain health and disease - related knowledge and consultations in a timely manner.	1	2	3	4	5
	5. Using mobile health services is beneficial for chronic disease management.	1	2	3	4	5
Perceived Ease of Use	6. Learning to use mobile health services is easy for me.	1	2	3	4	5
	7. The interface of mobile health services is clear and the operation is simple.	1	2	3	4	5
	8. I can use my smartphone alone to access mobile health services.	1	2	3	4	5
Use Intention	9. I am willing to use mobile health services.	1	2	3	4	5
	10. I am willing to recommend others to use mobile health services.	1	2	3	4	5
	11. I have the idea of using mobile health services.	1	2	3	4	5

移动医疗服务使用意愿量表

维度	题项	非常不同意	不同意	不确定	同意	非常同意
感知有用性	1. 使用移动医疗服务为我节省了时间	1	2	3	4	5
	2. 使用移动医疗服务使我的看病效率提高	1	2	3	4	5
	3. 对于敏感性的健康问题，使用移动医疗服务咨询医生更轻松	1	2	3	4	5
	4. 使用移动医疗服务有利于及时获得健康与疾病相关知识与咨询	1	2	3	4	5
	5. 使用移动医疗服务有利于进行慢性病管理	1	2	3	4	5
感知易用性	6. 学习移动医疗服务对我来说很容易	1	2	3	4	5
	7. 移动医疗服务界面清晰，操作简单	1	2	3	4	5
	8. 我可以独自使用智能手机获得移动医疗服务	1	2	3	4	5
使用意愿	9. 我愿意使用移动医疗服务	1	2	3	4	5
	10. 我愿意推荐别人使用移动医疗服务	1	2	3	4	5
	11. 我有使用移动医疗服务的想法	1	2	3	4	5
