# Supplementary material for: An Actor-Partner Interdependence Mediation Model for Assessing the Association Between Health Literacy and mHealth Use Intention in Dyads of Patients With Chronic Heart Failure and Their Caregivers: Cross-Sectional Study
Source: JMIR Mhealth Uhealth. 2025 Mar 6;13:e63805. doi: 10.2196/63805 (PMC11905925; doi:10.2196/63805)
Supplement: Multimedia Appendix 2 [file mhealth-v13-e63805-s002.pdf]

Results of the normality test

	Kolmogorov-Smirnov test			Shapiro—Wilk test		
	statistic	df	Sig.	statistic	df	Sig.
1	0. 081	312	＜0.001	0. 972	312	＜0.001
2	0. 095	312	＜0.001	0. 960	312	＜0.001
3	0. 122	312	＜0.001	0. 908	312	＜0.001
4	0. 153	312	＜0.001	0. 864	312	＜0.001
5	0. 202	312	＜0.001	0. 828	312	＜0.001
6	0. 345	312	＜0.001	0. 679	312	＜0.001
7	0. 234	312	＜0.001	0. 769	312	＜0.001
8	0. 383	312	＜0.001	0. 547	312	＜0.001

1: Chronic heart failure patients' health literacy

2: Caregivers' health literacy

3: Chronic heart failure patients' perceived usefulness of mHealth

4: Caregivers' perceived usefulness of mHealth

5: Chronic heart failure patients' perceived ease of use of mHealth

6: Caregivers' perceived ease of use of mHealth

7: Chronic heart failure patients' mHealth use intention

8: Caregivers' mHealth use intention
