# Supplementary material for: An Actor-Partner Interdependence Mediation Model for Assessing the Association Between Health Literacy and mHealth Use Intention in Dyads of Patients With Chronic Heart Failure and Their Caregivers: Cross-Sectional Study
Source: JMIR Mhealth Uhealth. 2025 Mar 6;13:e63805. doi: 10.2196/63805 (PMC11905925; doi:10.2196/63805)
Supplement: Multimedia Appendix 3 [file mhealth-v13-e63805-s003.pdf]

Multi-groups APIMeM

1. Patient Age Multi-groups APIMeM—Younger group—Perceived usefulness of mHealth

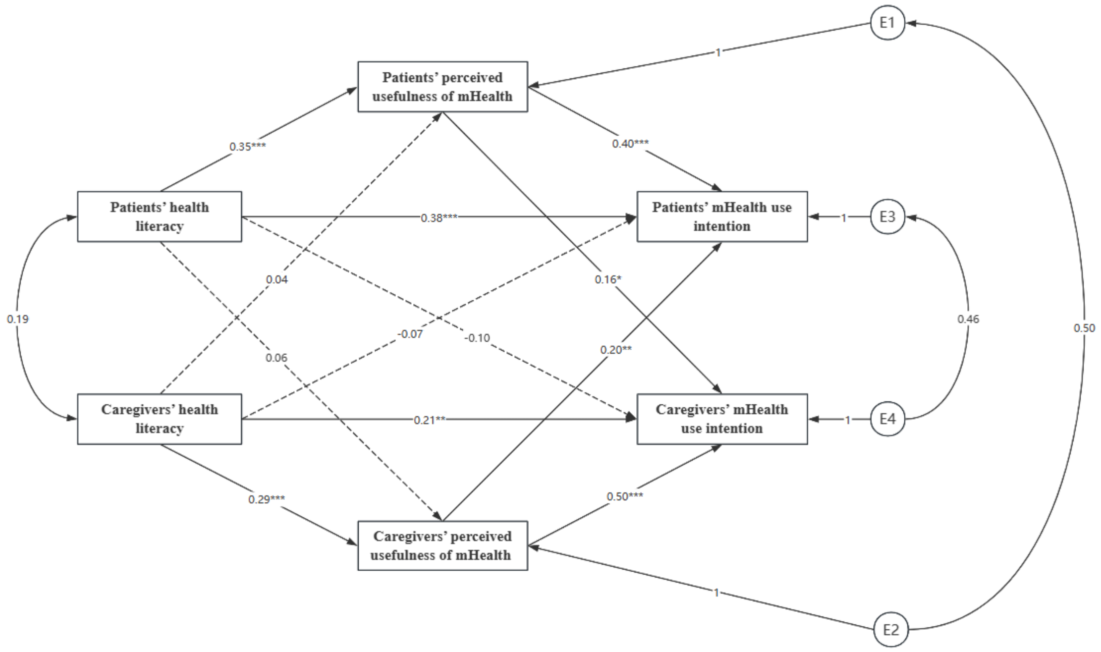

The model was a saturated model with 0 degrees of freedom, so its fit indices would no longer be estimated and only its path coefficients would be of interest.

2. Patient Age Multi-groups APIMeM—Older group—Perceived usefulness of mHealth

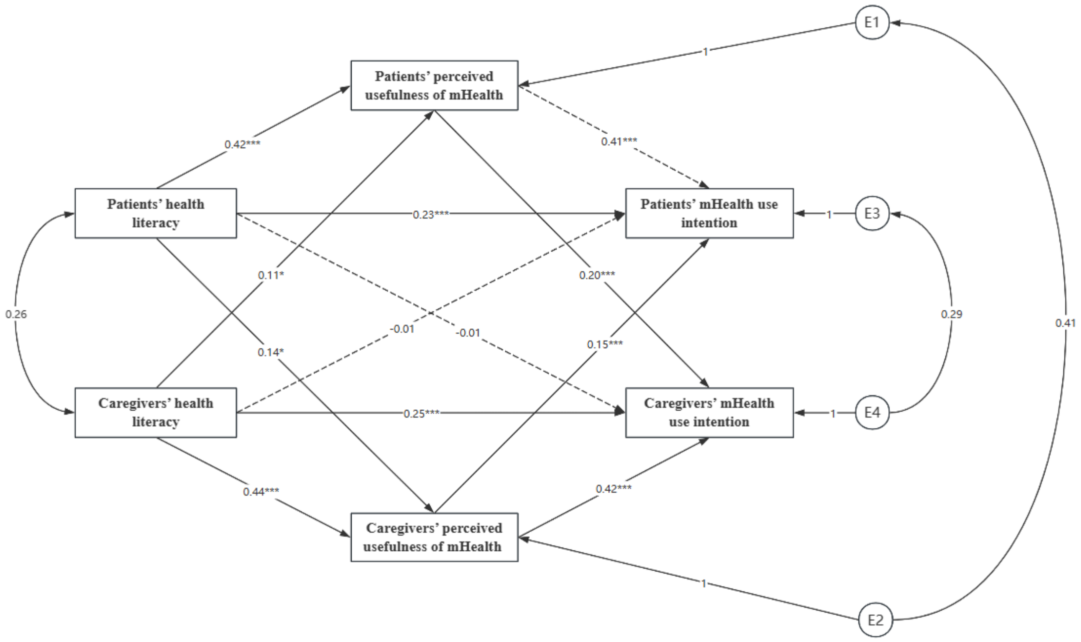

The model fit index $\chi2/df = 0.585$ (<3), RMSEA = 0.000 (≤0.08), CFI = 1.000 (≥0.90), TLI = 1.078 (≥0.90)

3. Patient Age Multi-groups APIMeM—Younger group—perceived ease of use of mHealth

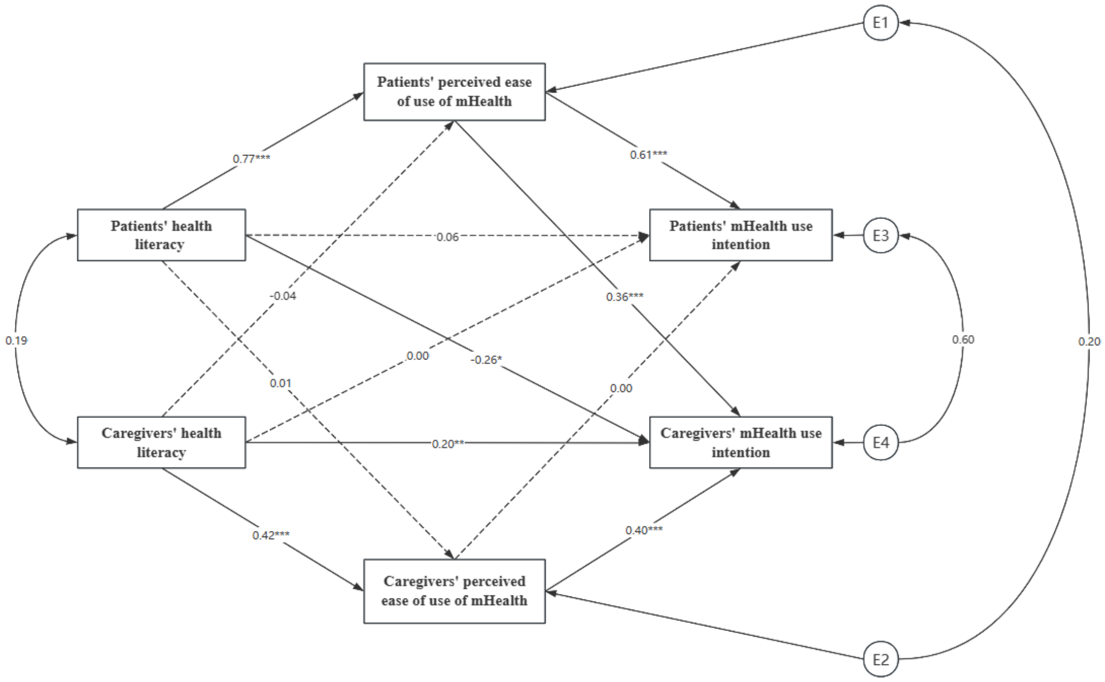

The model was a saturated model with 0 degrees of freedom, so its fit indices would no longer be estimated and only its path coefficients would be of interest.

4. Patient Age Multi-groups APIMeM—Older group—perceived ease of use of mHealth

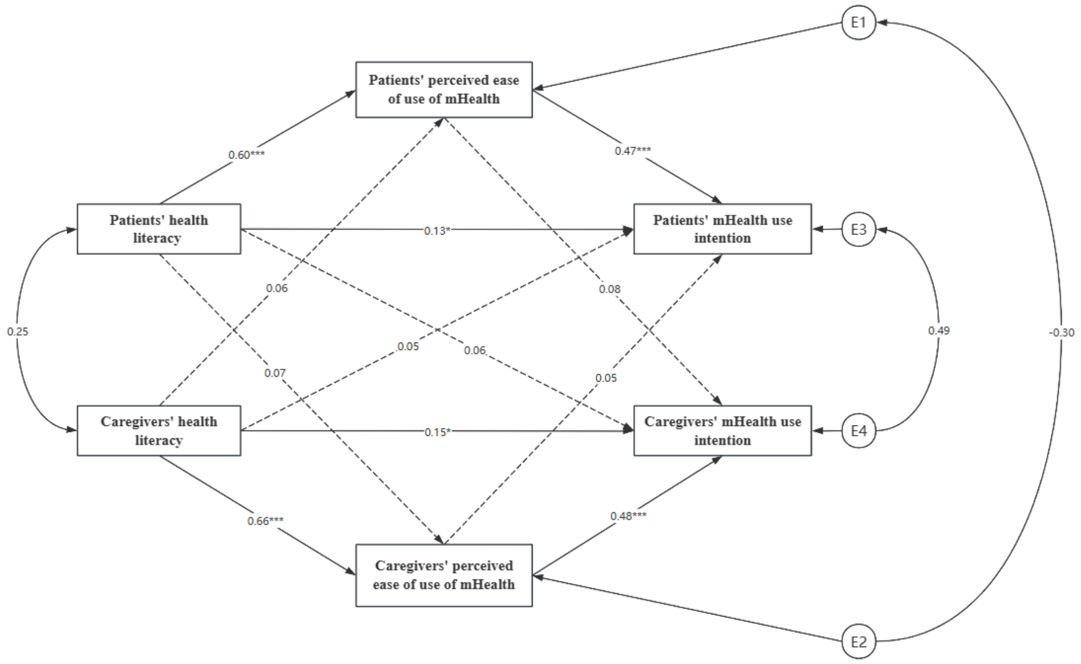

The model fit index χ2/df = 0.540 (<3), RMSEA = 0.000 (≤0.08), CFI = 1.000 (≥0.90), TLI = 1.059 (≥0.90)

5. Caregiver Age Multi-groups APIMeM—Younger group—Perceived usefulness of mHealth

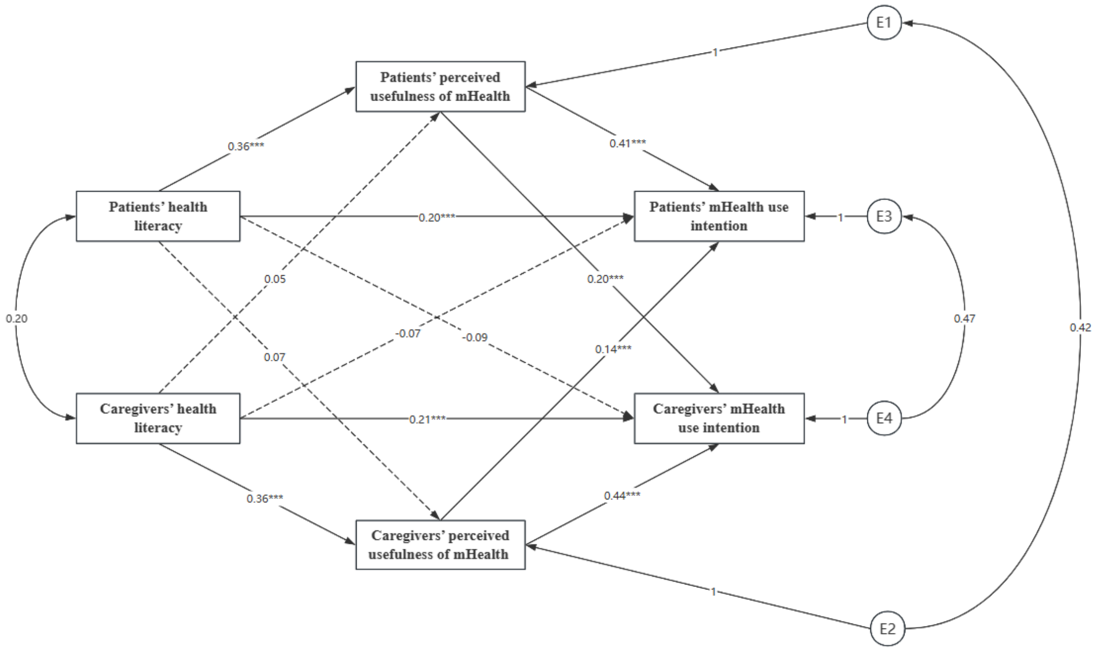

The model fit index χ2/df = 0.505 (<3), RMSEA = 0.000 (≤0.08), CFI = 1.000 (≥0.90), TLI = 1.075 (≥0.90)

6. Caregiver Age Multi-groups APIMeM—Older group—Perceived usefulness of mHealth

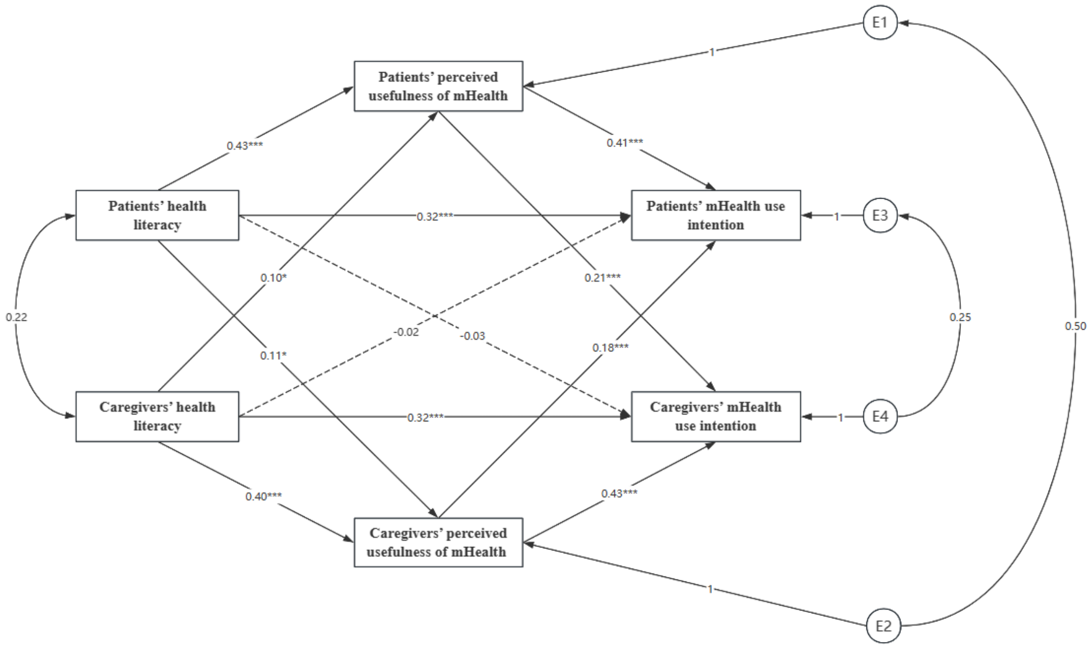

The model fit index χ2/df = 1.075 (<3), RMSEA = 0.022 (≤0.08), CFI = 0.995 (≥0.90), TLI = 0.988 (≥0.90)

7. Caregiver Age Multi-groups APIMeM—Younger group—perceived ease of use of mHealth

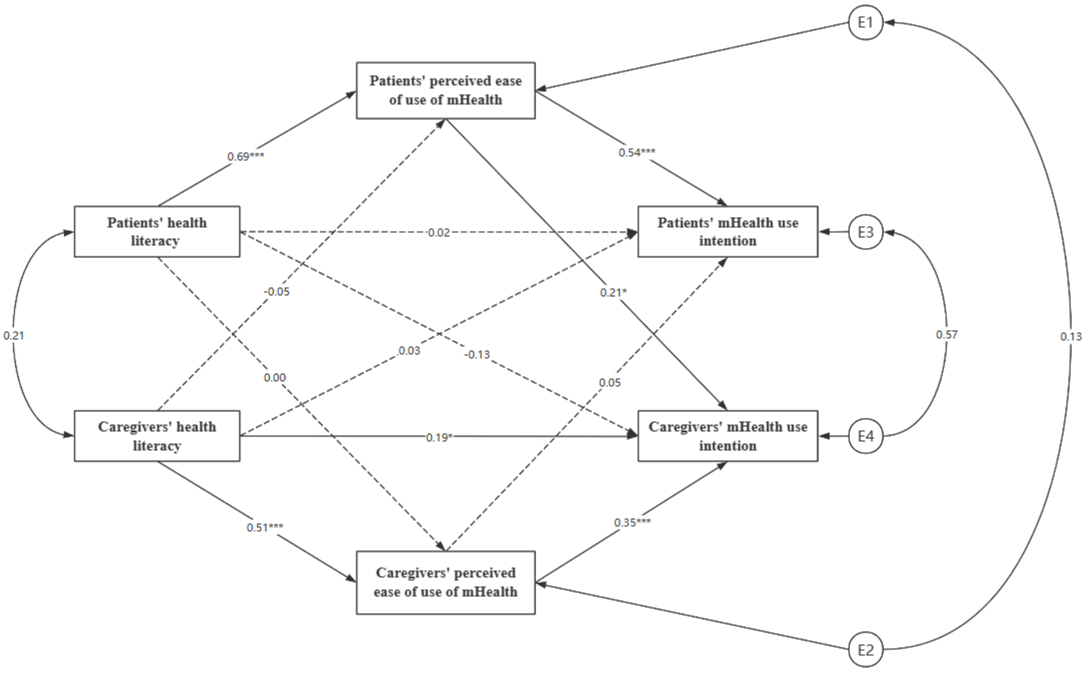

The model was a saturated model with 0 degrees of freedom, so its fit indices would no longer be estimated and only its path coefficients would be of interest.

8. Caregiver Age Multi-groups APIMeM—Older group—perceived ease of use of mHealth

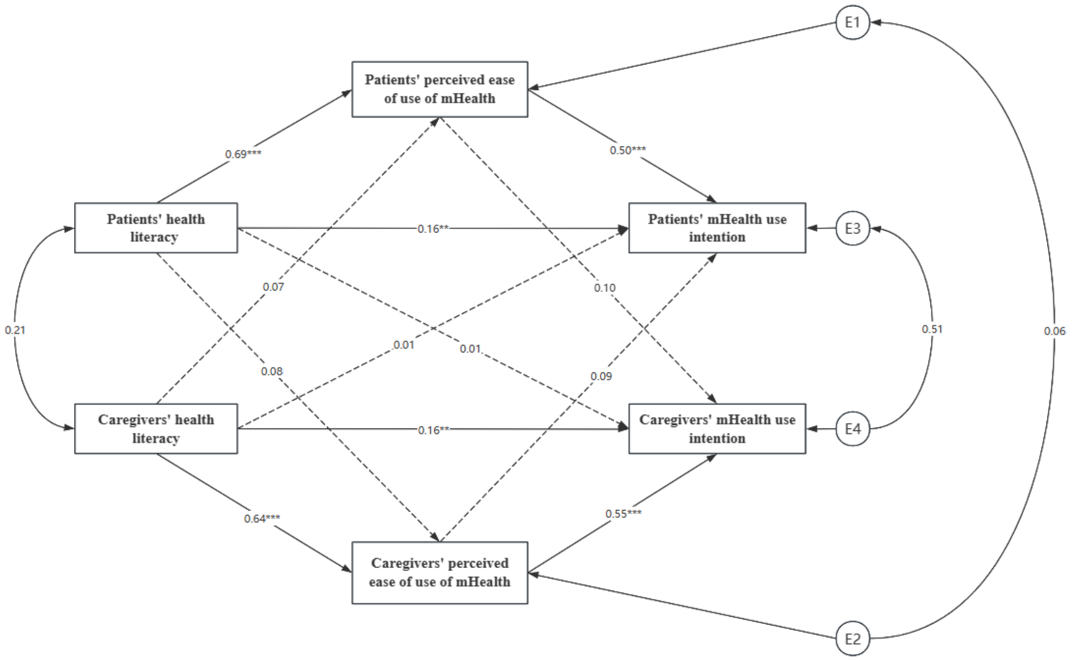

The model fit index χ2/df = 1.209 (<3), RMSEA = 0.037 (≤0.08), CFI = 0.990 (≥0.90), TLI = 0.975 (≥0.90)

9. Patient-Caregiver Relationships Multi-Group APIMeM—Couples Group—Perceived usefulness of mHealth

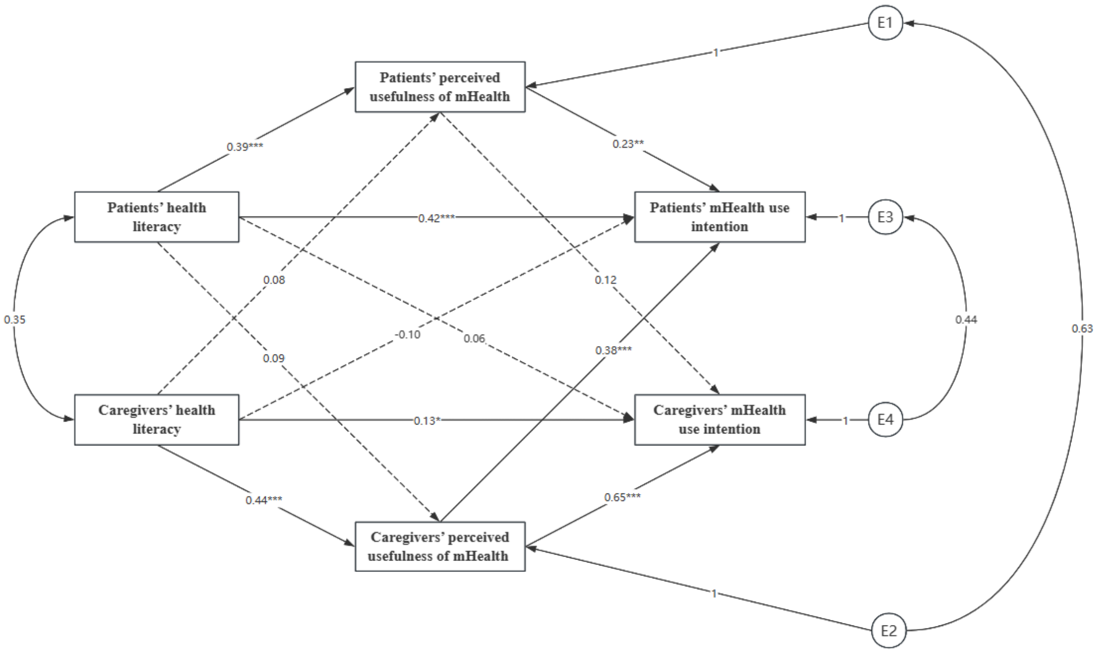

The model was a saturated model with 0 degrees of freedom, so its fit indices would no longer be estimated and only its path coefficients would be of interest.

10. Patient-Caregiver Relationships Multi-Group APIMeM—Son/daughter Group—Perceived usefulness of mHealth

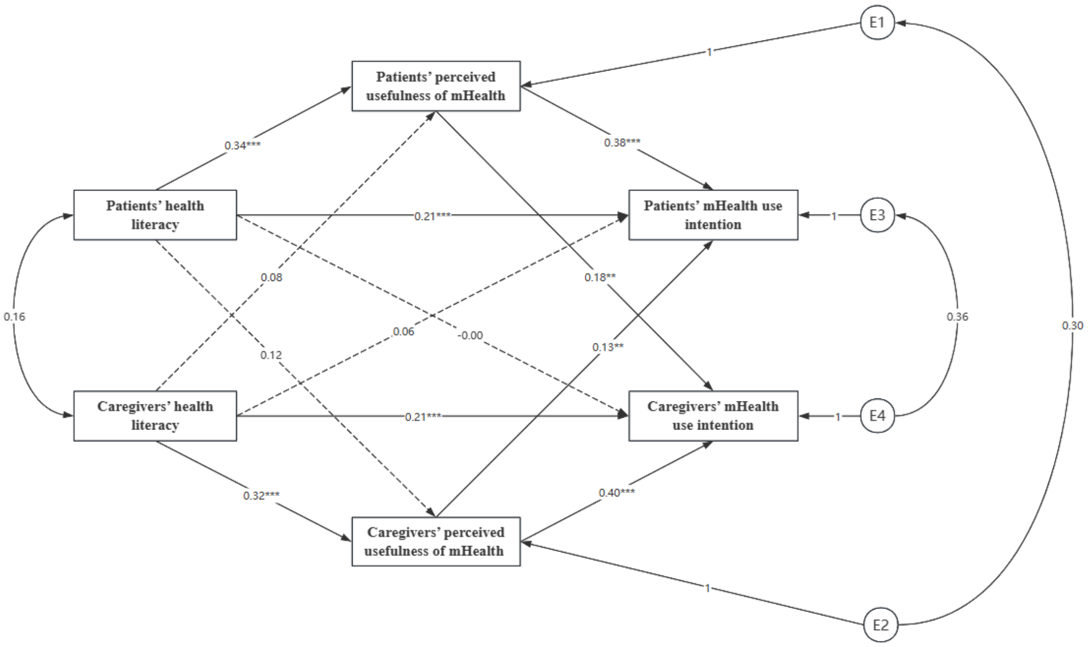

The model fit index $\chi 2/df = 0.991$ (<3), RMSEA = 0.000 (≤0.08), CFI = 1.000 (≥0.90), TLI = 1.002 (≥0.90)

11. Patient-Caregiver Relationships Multi-Group APIMeM—Other Group—Perceived usefulness of mHealth

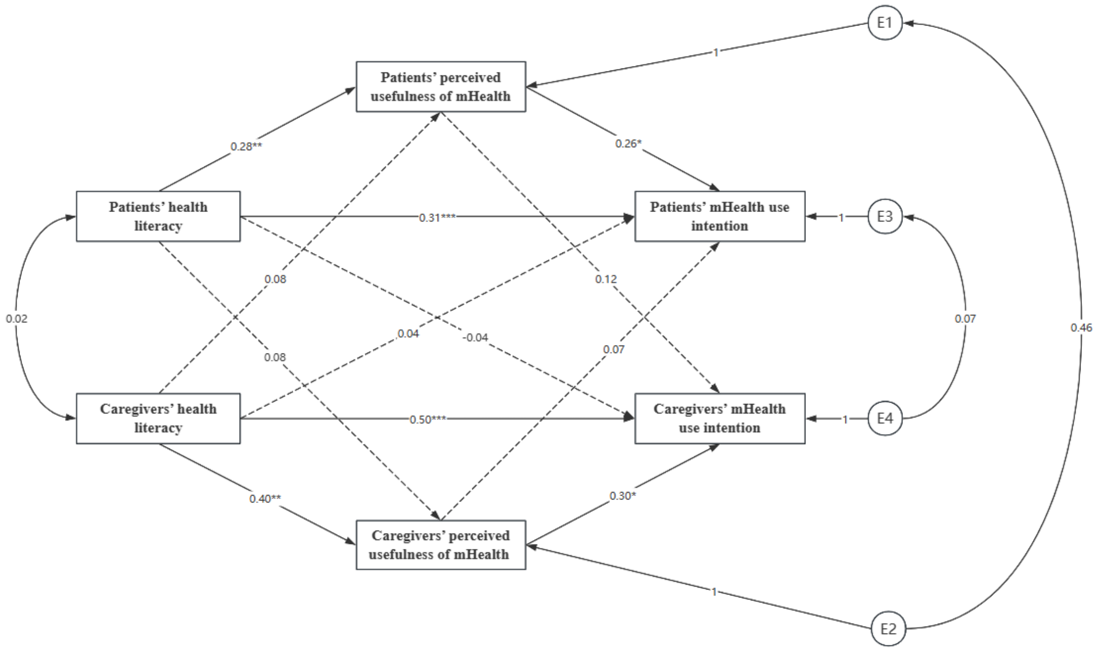

The model fit index χ2/df = 0.484 (<3), RMSEA = 0.000 (≤0.08), CFI = 1.000 (≥0.90), TLI = 1.881 (≥0.90)

12. Patient-Caregiver Relationships Multi-Group APIMeM—Couples Group—perceived ease of use of mHealth

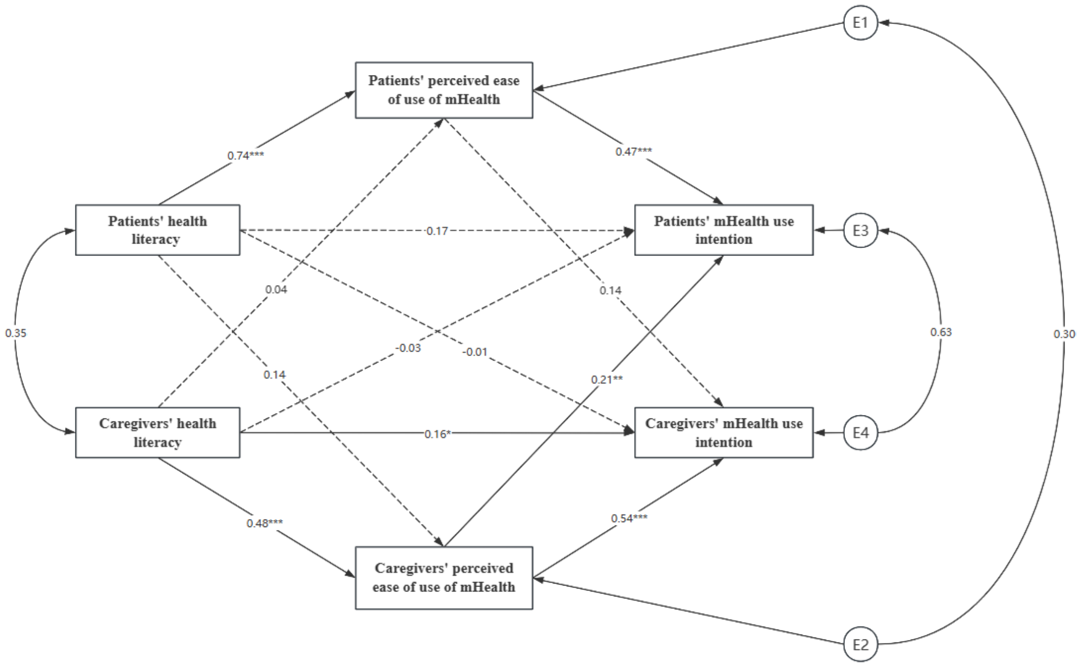

The model was a saturated model with 0 degrees of freedom, so its fit indices would no longer be estimated and only its path coefficients would be of interest.

13. Patient-Caregiver Relationships Multi-Group APIMeM—Son/daughter Group—perceived ease of use of mHealth

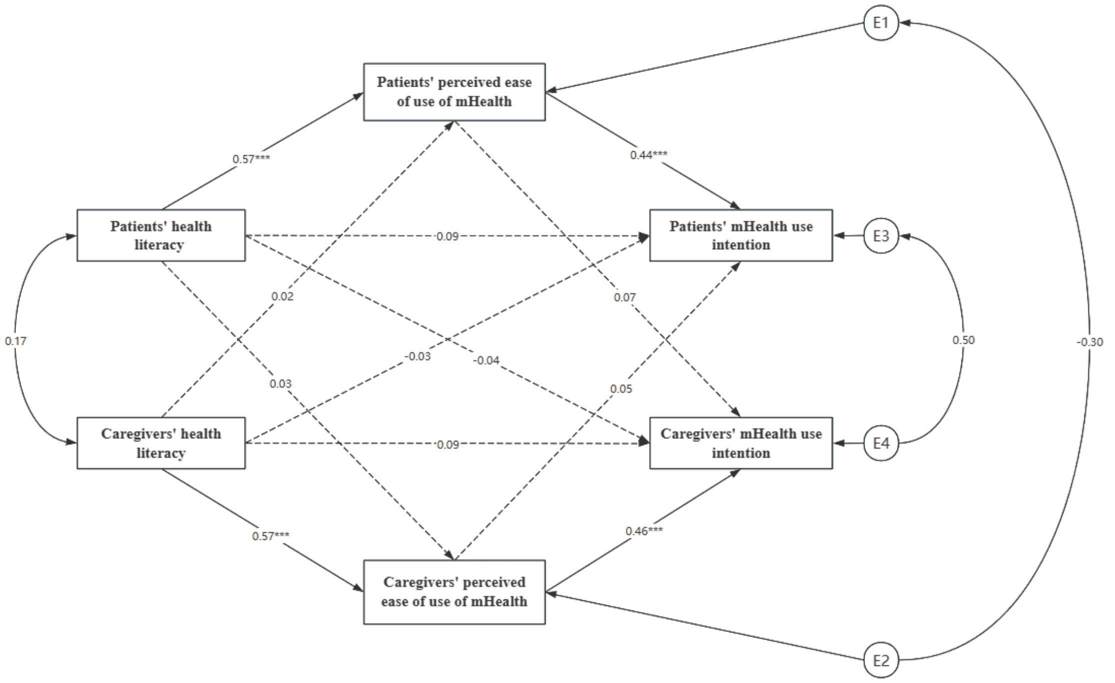

The model fit index χ2/df = 0.771 (<3), RMSEA = 0.000 (≤0.08), CFI = 1.000 (≥0.90), TLI = 1.034 (≥0.90)

14. Patient-Caregiver Relationships Multi-Group APIMeM—Other Group—perceived ease of use of mHealth

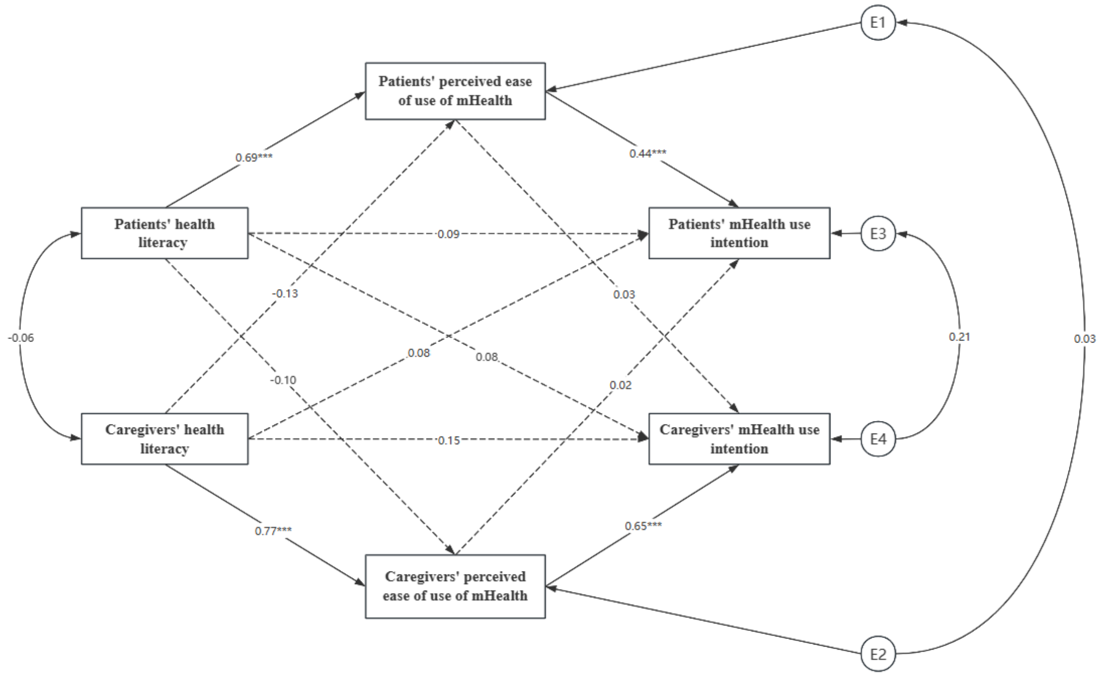

The model fit index χ2/df = 0.668 (<3), RMSEA = 0.000 (≤0.08), CFI = 1.000 (≥0.90), TLI = 1.293 (≥0.90)

Table 1 Test for differences in APIMeM path coefficients for multi-groups（t/Z/H, P）

APIMeM	Group	Normality test（P）	t/Z/H	P
Perceived usefulness of mHealth	Patient age-Younger<br>Patient age-Older	0.421	-0.366	0.718
Patient perceived ease of use of mHealth	Patient age-Younger<br>Patient age-Older	0.051	-0.243	0.810
Perceived usefulness of mHealth	Caregiver age-Younger<br>Caregiver age -Older	0.088	-0.665	0.513
Perceived ease of use of mHealth	Caregiver age -Younger<br>Caregiver age -Older	0.006	-0.722	0.470
perceived usefulness of mHealth	Relationship-Spouse<br>Relationship-Son/daughter<br>Relationship- Other	0.393	0.332	0.720
perceived ease of use of mHealth	Relationship-Spouse<br>Relationship-Son/daughter<br>Relationship- Other	0.001	1.021	0.600

Note.1. APIMeM: Actor Partner Interdependence Mediation model;

2. Perceived usefulness of mHealth: The actor-partner interdependent mediator model of health literacy-perceived usefulness of mHealth- mHealth use intention in chronic heart failure patients and caregivers;

3. Perceived ease of use of mHealth: The actor-partner interdependent mediator model of health literacy-Perceived ease of use of mHealth- mHealth use intention in chronic heart failure patients and caregivers;

4. Patient age-Younger: The data were ranked by age of the patients from youngest to oldest, with the upper half categorized as the younger group;

5. Patient age-Older: The data were ranked by age of the patients from youngest to oldest, with the bottom half categorized as the older group;

6. Caregiver age-Younger: The data were ranked by age of the caregivers from youngest to oldest, with the upper half categorized as the younger group.

7. Caregiver age -Older: The data were ranked by age of the caregivers from

youngest to oldest, with the bottom half categorized as the older group.

8. Relationship- Other: The data set for parent, friend and other kinship relationships.
